# An Unusual Case of Hypoxia: A Case of Right-to-Left Interatrial Shunting in a Patient With a Patent Foramen Ovale and Normal Pulmonary Pressure

**DOI:** 10.7759/cureus.22998

**Published:** 2022-03-09

**Authors:** Nardine Abdelsayed, Richard Duff, Mohamed Faris

**Affiliations:** 1 Department of Internal Medicine, Grand Strand Medical Center, Myrtle Beach, USA; 2 Department of Pulmonary and Critical Care Medicine, Grand Strand Medical Center, Myrtle Beach, USA; 3 Department of Internal Medicine, Grand Strand Regional Medical Center, Myrtle Beach, USA

**Keywords:** left heart cath, right heart cath, transthoracic echocardiogram, echocardiogram, right to left atrioventricular shunt, hypoxia, patent foramen ovale

## Abstract

A patent foramen ovale (PFO) is an embryological remnant. Hypoxia in the setting of a PFO is generally attributed to pulmonary hypertension resulting in an increase in right atrial pressure and mixing of venous blood from the right atrium with blood in the left atrium resulting in a right-to-left interatrial shunt (RLIAS), thus deoxygenating it. We present a case of a 64-year-old male with a past medical history of coronary artery disease (CAD) who presented with two weeks of dyspnea on exertion and intermittent chest pressure. He was found to be hypoxic at 87% (normal >95%) with largely normal workup except for left anterior descending (LAD) stenosis, which was stented, and a PFO that was found on transesophageal echocardiogram with normal pulmonary artery pressure (PAP). This case of hypoxia in the setting of a PFO without pulmonary hypertension puts into question the pathophysiology of hypoxia in a PFO and RLIAS.

## Introduction

A patent foramen ovale (PFO) is fairly common and occurs as an embryological remnant when the foramen ovale fails to close [1]. In the embryological stage, the foramen ovale creates a physiologic right-to-left interatrial shunt (RLIAS). Upon delivery, spontaneous ventilation results in an instant increase in pulmonary blood flow with an increased venous return to the left atrium increasing pressures on the foramen ovale, allowing for the fusion of the membranes which is composed of a septum primum and secundum. A deficiency in portions of this septum results in a PFO. 

Although generally asymptomatic, PFOs have been associated with a variety of medical disorders including cerebrovascular accidents, transient ischemic attacks, migraines, decompression syndrome, and systemic desaturation. Initially, a PFO provides left-to-right interatrial shunting since the left atrial pressure is greater than that of the right atrium. Eventually, some patients may develop Eisenmenger syndrome resulting in RLIAS allowing for deoxygenated blood returning to the right atrium to shunt through the left atrium and is ejected into the systemic circulation. Cases like ours highlight the occurrence of hypoxia and RLIAS in a patient without pulmonary hypertension, suggesting an alternative mechanism of hypoxia in RLIAS.

The five main mechanisms of hypoxia are hypoventilation, ventilation-perfusion mismatch, right-to-left shunts (may be anatomic or physiologic), diffusion limitations, and reduced inspired oxygen tension [2]. Hypoventilation may occur in the setting of central nervous system (CNS) depression, obesity hypoventilation, impaired neuronal conduction, muscular weakness (such as in myasthenia gravis or severe hypothyroidism), or poor chest wall elasticity. CNS depression may occur in the setting of drug overdose or structural CNS lesions. Poor chest wall elasticity may be seen in conditions such as kyphoscoliosis and flail chest. Ventilation-perfusion (V/Q) mismatch generally occurs when there is an imbalance of the pulmonary blood supply and ventilation. Hypoxia that is due to V/Q mismatch generally improves with supplemental oxygen. Conditions that cause this include obstructive lung disease, interstitial disease, emphysema, heart failure, or pulmonary vascular disease such as pulmonary emboli. Right-to-left shunt is discussed above and occurs when blood travels from the right to left atrium without being oxygenated, causing severe V/Q mismatch. Anatomic shunts may include atrial septal defects, PFOs, pulmonary arteriovenous malformations, and hepatopulmonary syndrome. Physiologic shunts include atelectasis and alveolar filling diseases, such as with red blood cells, white blood cells, fluid, protein, or other tissue cells. Diffusion limitation occurs when the alveoli are inflamed or fibrose. Reduced oxygen-inspired tension occurs in areas of reduced oxygen saturation, such as at elevated altitudes. In the setting of hypoxia, there is insufficient oxygen for cells to meet their metabolic demands. 

Hypoxia is diagnosed by pulse oximetry and generally presents with dyspnea on exertion. The past medical history is an important component in determining the cause and should include the history of pulmonary or cardiac disease. A thorough history also includes current medications, focusing on opioids and benzodiazepines, as well as illicit drugs. Any active symptoms should be addressed. Conditions that cause impaired neuronal conduction such as amyotrophic lateral sclerosis, Guillain-Barre syndrome, cervical spine injury, phrenic nerve paralysis can all result in hypoxia and should be examined [2]. A patient's weight could be an indicator of possible obesity hypoventilation syndrome or obstructive sleep apnea. Physical exam should pay special attention to the chest wall, examining for kyphosis or flail chest, and pulmonary exam, which may demonstrate crackles or wheezing. Laboratory studies should include arterial blood gasses, comprehensive blood count, comprehensive metabolic panel, and thyroid function tests. A chest x-ray and echocardiogram should be obtained. An echocardiogram can show an atrial septal defect or PFO as well as assess pulmonary artery pressure to determine the presence of pulmonary hypertension. A V/Q scan or computed tomography angiography (CTA) chest can also be obtained if there is a concern for pulmonary embolism.

## Case presentation

A 64-year-old male with a past medical history of coronary artery disease (CAD) presented to our institution with progressive dyspnea on exertion and intermittent chest pressure of two weeks duration. His medical history was notable for CAD with stent placement to the left anterior descending artery (LAD) six months prior to presentation and obstructive sleep apnea. Social history was notable for his job in coal mining for two years. He was a non-smoker. 

**Figure 1:**
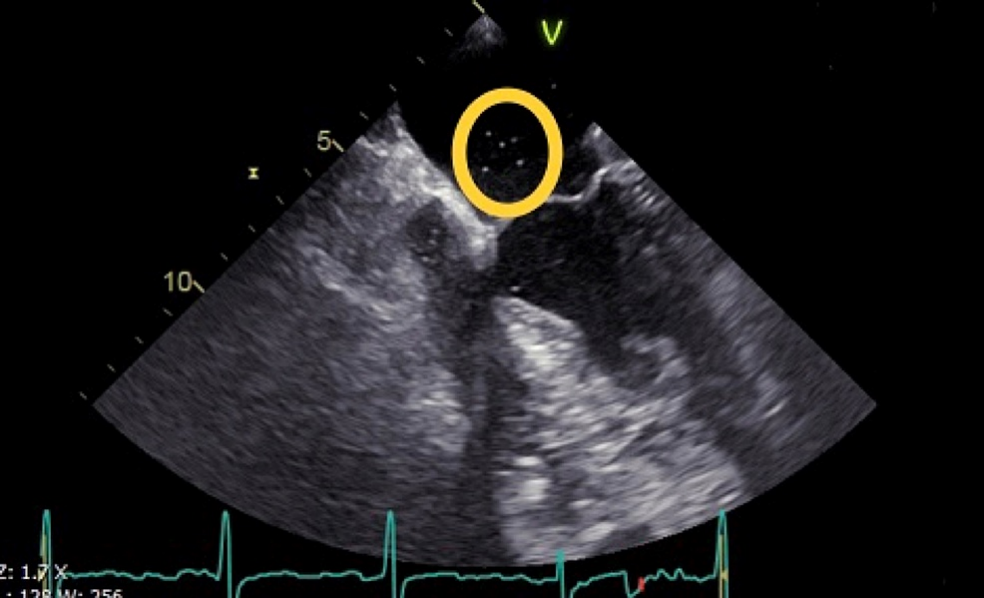
Transesophageal Echocardiogram (TEE) with yellow circle showing the RLIAS with evidence of PFO RLIAS: right-to-left interatrial shunt; PFO: patent foramen ovale

On admission, his vital signs were normal except for his oxygen saturation, which was 87% on room air. Physical examination did not reveal any abnormalities. Laboratory investigations were unremarkable with normal hemoglobin and hematocrit levels. A computer tomography angiography (CTA) of the chest did not reveal evidence of pulmonary embolism or other abnormalities. Ventilation-perfusion scan showed normal perfusion. Pulmonary function tests were normal. Right and left heart catheterization revealed critical proximal LAD stenosis, normal cardiac output, and index by thermodilution. Findings are seen in Table 1 and revealed normal pulmonary capillary wedge pressure (PCWP), left ventricular end-diastolic pressure (LVEDP), and pulmonary artery pressure (PAP). A drug-eluting stent (DES) was placed in the LAD. A transthoracic and transesophageal echocardiogram with agitated saline showed evidence of a PFO with right-to-left shunting by evidence of color flow Doppler (Figure 1) with an enlarged right atrium and right ventricle. 

**Table 1:** Measured pressures during right heart catheterization

	Measured value	Normal range
Pulmonary capillary wedge pressure (PCWP)	12 mmHg	4-12 mmHg
Left ventricular end-diastolic pressure (LVEDP)	12 mmHg	4- 12 mmHg
Pulmonary artery systolic pressure	32 mmHg	8-25 mmHg
Pulmonary artery diastolic pressure	15 mmHg	8-15 mmHg
Mean pulmonary artery pressure	22 mmHg	< 20 mmHg (elevated when >25 mmHg at rest)
Right atrial pressure	10 mmHg	2-6 mmHg
Right ventricle systolic pressure	13 mmHg	20-30 mmHg
Right ventricle diastolic pressure	10 mmHg	3-7 mmHg

The patient’s hypoxia was attributed to RLIAS in the absence of other causative factors. He underwent successful closure of the PFO with subsequent resolution of hypoxia.

## Discussion

Our workup was negative except for a PFO and RLIAS. RLIAS in PFOs generally occurs when chronic left-to-right shunting results in pulmonary hypertension increasing right atrial pressures until they rise above left atrial pressures, a condition called Eisenmenger syndrome. Since our patient did not have pulmonary hypertension, this put into question the mechanism of RLIAS in his PFO. One proposed theory of RLIAS in patients with PFO without evidence of pulmonary hypertension is the presence of a remnant eustachian valve (EV). One study found that 70% of patients with PFO continue to have an EV, which is a remnant of the embryonic right valve of the sinus venosus. In fetal circulation, the EV is physiologic and directs oxygenated blood from the inferior vena cava across the PFO and into the systemic circulation. If present in an adult, this may allow for preferential blood flow towards the PFO and resultant RLIAS [3]. RLIAS may also occur in the setting of certain anatomical abnormalities in the absence of pulmonary hypertension such as pneumonectomy [4], diaphragm paralysis, kyphoscoliosis, the elevation of the right hemidiaphragm [5], dilation of the ascending aorta [5], and obstructive sleep apnea. Platypnea-orthodeoxia can also occur in patients with PFO and presents as dyspnea and hypoxia that occur when patients change from a supine to an upright position [6,7]. This is due to the physiological increase in right atrial pressure in the setting of a change in position or Valsalva maneuver, causing a reversal in blood flow and a transient RLIAS ultimately resulting in hypoxia. 

PFOs are diagnosed using an echocardiogram with bubbles or with contrast. In a bubble study, agitated saline is first injected into the body during the study. The study is positive when contrast is noted to be in the left atrium during early ventricular systole. A transesophageal echocardiogram (TEE) is then performed to confirm the PFO as well as to characterize its anatomy and dimensions, in order to assess its suitability for device closure. 

Indications for PFO closure include cryptogenic strokes with a high risk of paradoxical embolism (RoPE) scores, decompression syndrome, platypnea-orthodeoxia syndrome, and shunt-induced cyanosis. In patients with a PFO who present with a cryptogenic stroke, the RoPE score is a convenient tool for estimating the probability that a stroke is associated with a PFO, rather than it being an incidental finding. A score greater than 7 indicates a high probability of association. One important contraindication of PFO closure is irreversible pulmonary hypertension due to the risk of decompensation of the right ventricular function and a resultant drop in cardiac output after closure [8]. Generally, percutaneous closure is implemented unless other defects are present that require intervention. It is generally safe, but like all procedures has risks including arrhythmias, infection, device migration, or hemorrhage in the setting of a traumatic puncture of the coronary vessels. One study found in patients with a PFO who have normal right-sided pressures, percutaneous PFO closure may result in marked improvement of their symptoms [9].

## Conclusions

PFOs are common in the general population and may result in marked hypoxia in the setting of RLIAS. The pathophysiology of RLIAS in PFOs generally occurs in the setting of pulmonary hypertension and increased right atrial pressure in a condition called Eisenmenger syndrome. Our patient with a PFO and a normal pulmonary artery pressure (PAP) with a negative workup for other causes of hypoxia suggests alternative pathophysiology that is less well understood. Normal pulmonary pressures in these patients, therefore, should not dissuade further intervention with percutaneous PFO closure in patients with hypoxia since closure has shown to improve both hypoxia and quality of life.
